# Development and application of a biomarker assay for determining the pharmacodynamic activity of an antagonist candidate biotherapeutic antibody to IL21R in whole blood

**DOI:** 10.1186/1479-5876-8-51

**Published:** 2010-05-28

**Authors:** Maya Arai, Sadhana Jain, Amy A Weaver, Andrew A Hill, Yongjing Guo, Andrea G Bree, Michael F Smith, Scott W Allen, Edward R LaVallie, Deborah Young, Laird Bloom, Karissa Adkins, Margot O'Toole

**Affiliations:** 1 Global Biotherapeutic Technologies, Pfizer, 87 Cambridge Park Drive, Cambridge, MA 02140, USA; 2 Massachusetts Research Business Technologies, Pfizer, 35 Cambridge Park Drive, Cambridge, MA 02140, USA; 3 Inflammation and Immunology, Pfizer, 200 Cambridge Park Drive,Cambridge, MA 02140, USA; 4 Translational Medicine- Inflammation, Hoffmann-LaRoche, 340 Kingsland St., Nutley, NJ 07110, USA; 5 Investigative Toxicology, Pfizer, 1 Burtt Road, Andover, MA 01810, USA; 6 Drug Safety Research and Development, Eastern Point Road, MS8274-1222, Groton, CT 06340, USA; 7 Translational Medicine, BioTherapeutic Research, Pfizer, 35 Cambridge Park Drive,Cambridge, MA 02140, USA

## Abstract

**Background:**

In preparation for potential clinical development of Ab-01, an antagonistic antibody directed against the IL21R, studies were undertaken to address translational medicine needs that fall into four categories: 1) development of a pharmacodynamic biomarker assay suitable for use in the clinic, 2) demonstration that Ab-01 has the desired biological activity *in vitro *and *in vivo *in cynomolgus monkeys, the preferred safety study species, 3) pre-clinical *in vivo *proof-of-concept that the assay can be used to detect Ab-01 pharmacodynamic (PD) activity in treated subjects, and 4) comprehensive assessment of the agonistic potential of Ab-01 when cross-linked. This report and a recently published companion report address the first three of these needs. The fourth has been addressed in a separate study.

**Methods:**

Genes that change RNA expression upon *ex vivo *rhIL21 stimulation of whole blood were identified in human and cynomolgus monkey. The inhibitory effects of exogenously added Ab-01 were measured *ex vivo *in human and monkey, and the *in vivo *inhibitory effects of Ab-01 treatment were measured in monkey.

**Results:**

Stimulation of whole human blood for 2 hours with rhIL21 induced robust increases in RNA expression of 6 genes. This response was blocked by Ab-01, indicating that the assay is suitable for measuring Ab-01 activity in blood. rhIL21 induced expression of a similar set of genes in cynomolgus monkey blood. This response was blocked with Ab-01, thus demonstrating that Ab-01 has the desired activity in the species, and that safety studies done in cynomolgus monkeys are relevant. Proof -of-concept for using this assay system to detect PD activity *in vivo *was generated by measuring the response in monkey blood to *ex vivo *rhIL21 stimulation before and 5 minutes following *in vivo *Ab-01 administration.

**Conclusions:**

A robust PD biomarker assay suitable for clinical use has been developed in human whole blood. The successful adaptation of the assay to cynomolgus monkeys has enabled the demonstration of Ab-01 activity both *in vitro *and *in vivo *in monkey, thus validating the use of this species in safety studies and establishing proof-of-concept for using this PD assay system to aid in dose selection in clinical studies.

## Background

Development of protocols for appropriate dose selection in clinical studies is a clear priority within medical [1] and regulatory [2] communities. The high attrition rate of drugs in development due to toxicity and/or lack of efficacy [3,4] underscores the need for biomarker assays to provide early information on whether the compound being tested does indeed have the expected effect on the targeted pathway. This information can be used to mitigate the risk of entering into lengthy and expensive efficacy studies. To have an impact on clinical development, a robust PD biomarker assay must be developed well in advance of phase I clinical studies. The assay must also function reliably in the population used for phase I studies, which, in the case of compounds directed towards blockade of inflammatory pathways, is often a healthy volunteer population. To develop biomarkers for drugs targeting inflammatory pathways, previous investigators have turned to *ex vivo *stimulation in whole blood [5,6]. This approach has been particularly useful in the development of p38 MAPK inhibitor compounds [7] in which LPS (lipopolysaccharide)-induced production of inflammatory cytokines can be measured. We followed this basic approach (*ex vivo *stimulation of whole blood) to develop pharmacodynamic biomarker assays for a candidate therapeutic antibody, Ab-01.

Ab-01, a human antibody generated by phage display, recognizes the high affinity receptor for IL-21, IL21R, blocks IL21-mediated immune activation through antagonist engagement of IL21R and has shown efficacy in a mouse model of lupus [8]. The goal of the biomarker strategy was to provide the means of avoiding toxicity due to unnecessarily high drug levels and lack of efficacy due to ineffective dosing by providing early clinical data on how well the drug hits the target *in vivo, *and on the best dosing regimen to maintain target engagement/inhibition. A second critical goal while preparing for potential clinical testing was clear demonstration of the desired biological activity in cynomolgus monkeys, the safety study species. In the absence of such data, the relevance of safety studies is uncertain. Therefore, in parallel, we applied our biomarker strategy to cynomolgus monkeys and used it to examine *ex vivo *and *in vivo *Ab-01 activity in this species. Here we report the development of PD biomarker assays that measure Ab-01 biological activity in human and cynomolgus monkey samples. In addition we provide pre-clinical proof-of-concept that the assay system can be used to measure PD activity in treated subjects.

## Methods

### Sample source and human PD biomarker assay development

Pilot studies on whole blood from 12 healthy human donors were performed to identify biomarkers of *ex vivo *response of blood to stimulation with rhIL21. Human blood samples from healthy volunteers were collected under the Wyeth Human Blood Donor Program - a program approved and administered by Mt Auburn Hospital, Cambridge, MA. Informed consent was obtained from all donors. A total of 7 donors were used for the initial pilot studies used for assay development, and an additional 9 donors were used for the confirmatory experiments reported here. Whole blood samples were collected in BD Vacutainer™ CPT™ cell preparation tubes containing sodium heparin (Catalogue #362753). For all data shown samples were maintained at ambient temperature and were processed within an hour of collection, but additional studies indicated acceptable assay performance in blood that had been stored overnight at room temperature (data not shown).

### Protein reagents: rhIL-21, Ab-01, and control antibodies

The protein reagents used in this study - rhIL21 (recombinant human IL21), anti-IL21 receptor antibody Ab-01 (also known as clone VL6 and ATR-107), control antibody human IgG1 α-tetanus triple mutant (IgG_1_TM, containing the same mutations in the Fc region as Ab-01), were made by the Biological Technologies Department at Wyeth (now Pfizer) Research (Cambridge, MA). Characteristics of rhIL21 are described in Additional file 1. The three mutations common to the Fc portion of Ab01 and IgG1TM reduced their potential effector activity. Antibodies with these mutations had undetectable activity in antibody-dependent cell-mediated cytotoxicity (ADCC) or C1q binding assays [9,10]. An antibody with severely compromised effector function was chosen for development because the therapeutic goal is to block the interaction of IL21 with IL21R, and therefore minimization of effector function is desirable. Endotoxin levels in all proteins reagents were determined to be below 1.0 EU/mg.

### *Ex vivo *treatment of human blood

Human blood was distributed (1 mL/aliquot) into screw cap cryovials (Nunc, Cat# 375353). All treatments were run in duplicate. rhIL21 (produced from Chinese hamster ovary cells at Wyeth, now Pfizer) was added in volumes ranging from 3 μL to 10 μL to achieve the indicated concentration. A similar volume of PBS was added to unstimulated control samples. Samples were incubated at 37°C for the indicated duration while mixing continuously at approximately 15 revolutions per minute using a Rotamix rotating mixer (ATR Inc, Laurel, MD). To investigate Ab-01-mediated inhibition of rhIL21 response, Ab-01 was added to blood prior to addition of rhIL21. During the assay development phase of the work, Ab-01 was added immediately prior to addition of rhIL21, and total inhibition of the response was observed (data not shown). Since manipulation of samples immediately upon collection would not have been practical in the setting of a clinical study, the final assay protocol included a two hour incubation period in the presence or absence of Ab-01. This protocol mimicked the conditions of the intended clinical use of the assay, since blood from Ab-01 treated subjects (containing Ab-01) would have to placed in a queue in a laboratory prior to addition of rhIL21. The experiments with human blood reported here included a 2 hour incubation at 37°C prior to the addition of rhIL21. Human blood (in 1 mL aliquots) was pre-incubated for 2 hours with Ab-01 or IgG_1_TM control immunoglobulin at increasing concentrations followed by the addition of rhIL21(10 ng/mL) and subsequent 2 hour incubation.

### RNA isolation

Aliquots of blood (0.5 mL) were removed following treatments and added to 2.0 mL microtubes (Axygen Scientific, Union City, CA) containing 1.3 mLs of RNA*later^® ^*(Applied Biosystems/Ambion, Austin, TX, Catalogue #AM1928), and mixed thoroughly by 5 complete inversions. Samples were stored at ambient temperature overnight and then frozen at -80°C pending RNA purification. RNA was isolated using the Human RiboPure™-Blood Kit (Applied Biosystems/Ambion Austin TX, Catalogue #AM1928) following the manufacturer's protocol. The Human RiboPure™ RNA isolation procedure involves cell lysis in a guanidinium-based solution and initial purification of the RNA by phenol/chloroform extraction followed by final RNA purification by solid-phase extraction on a glass-fiber filter. The residual genomic DNA was removed according to the manufacturer's instructions by DNAse treatment using the DNA-*free*™ reagents provided in the kit.

RNA quantity was determined by absorbance at 260 nm with a NanoDrop 1000 (NanoDrop, Wilmington, DE). RNA quality was evaluated using a 2100 Bioanalyzer (Agilent, Palo Alto, CA, Agilent 2100 expert software version B.02.05.SI360), and all samples had RIN (RNA integrity number) [11] >6.6, and all but 2 had RIN values >7.0. Samples were stored at -80°C until cDNA synthesis was performed.

### Measurement of gene expression levels using real time RT-PCR

Based on results from the pilot studies (data not shown), assays for gene transcripts with potential as biomarkers were selected for inclusion on a custom TaqMan Low Density Array (TLDA) purchased from Applied Biosystems (ABI) Foster City, CA. This TLDA contained a total of 24 assays measuring 19 potential biomarkers and 5 endogenous controls (Table 1). Two independent measurements of each transcript were obtained from each sample. Following the manufacturer's instruction, 400 ng of total RNA were used to generate cDNA in 40 μL reaction volume in a DNA Engine Peltier Thermal Cycler (MJ Research, GMI Inc., Ramsey, MN) using a High Capacity cDNA Reverse Transcription Kit (ABI, #4368814) with addition of RNase Inhibitor at 50 U/sample (ABI, #N808-0119). Reaction conditions were: 25°C for 10 minutes, 37°C for 2 hours, 85°C 5 seconds and then hold at 4°C. If TLDA amplification reactions were not performed on the same day as cDNA synthesis, the cDNA samples were stored at -20°C. The amount of cDNA to be loaded on the TLDA was determined empirically by titration in a pilot study. Results showed that the amount of cDNA produced from 200 ng of starting RNA yielded values above the lower detection limit for all but two of the candidate biomarkers, and 200 ng (equivalent) was used in all subsequent experiments. The cDNA product (in 20 μl volume) was diluted by addition of 30 μl DEPC water and mixed with 50 μl TaqMan^® ^Universal 2 × PCR Master Mix (ABI, #4304437) for a final volume of 100 μl, and added to each TLDA port. Assay was performed on an ABI PRISM 7900 Sequence Detector (Sequence Detector Software v2.2.2) using universal thermal cycling conditions of 50°C for 2 minutes, 95°C for 10 minutes, followed by 40 cycles of 95°C for 15 seconds and 60°C for 1 minute. Data output was generated from ABI's SDS 2.2.2 software that determines C_T _(threshold cycle) values from the PCR amplification plot.

**Table 1:** Assays used to measure human genes on custom TaqMan low density array for human studies

*Gene*	*Gene Description*	*Assay ID (ABI)*
18S*	Eukaryotic 18S rRNA	Hs99999901_s1
*CCL3*	chemokine (C-C motif ligand	Hs00234142_m1
*CD19*	CD19	Hs00174333_m
*CXCL10*	chemokine (C-X-C motif ligand 10)	Hs00171042_m1
*CXCL11*	chemokine (C-X-C motif ligand 11)	Hs00171138_m1
*GNLY*	Granulysin	Hs00246266_m1
GAPDH*	glyceraldehyde 3 phosphate dehydrogenase	Hs99999905_m1
GUSB*	glucuronidase, beta	Hs99999908_m1
*GZMB*	Granzyme B (cytotoxic T lymphocyte-associated serine esterase 1)	Hs00188051_m1
*ICAM1*	intercellular adhesion molecule 1 (CD54)	Hs00164932_m1
*IFNg*	interferon, gamma	Hs00174143_m1
*IL10*	interleukin 10	Hs00174086_m1
*IL12A*	interleukin 12A (natural killer cell stimulatory factor 1	Hs00168405_m1
*IL1b*	interleukin 1, beta	Hs00174097_m1
*IL21R*	interleukin 21 receptor	Hs00222310_m1
*IL2RA*	interleukin 2 receptor, alpha, CD25	Hs00166229_m1
*IL6*	interleukin 6	Hs00174131_m1
*IL8*	interleukin 8	Hs00174103_m1
PGK1*	phosphoglycerate kinase	Hs99999906_m1
*PRF1*	perforin 1 (pore forming protein)	Hs00169473_m1
*STAT3*	signal transducer and activator of transcription 3	Hs00234174_m1
*TBX21*	T box 21	Hs00203436_m1
*TNF*	tumor necrosis factor (TNF superfamily, member 2)	Hs00174128_m1
ZNF592*	zinc finger protein 592	Hs00206029_m1

*Gene used as endogenous normalizer

### Description of calibrators and normalization of results using endogenous control genes

**Figure 1:**
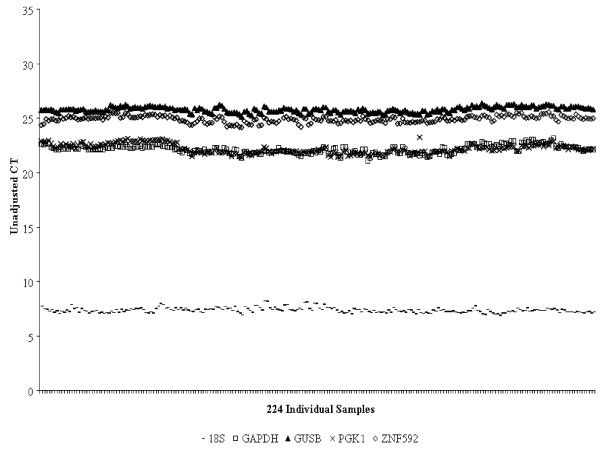
**Expression levels of normalizer genes**. The unadjusted C_T _values for 5 genes used as endogenous normalizers are shown and reveal very similar levels of expression in all study samples.

Calibrator samples functioned as the common comparator for RQ (Relative Quantification of RNA expression) calculations. The average C_T _values for all genes in the unstimulated samples from the first 5 donors following 2 hour incubation served as calibrator for experiments used to determine optimal rhIL21 dose and time course. Similarly, the average C_T _of the unstimulated samples from the second set of 4 donors was used as calibrator for the experiments related to titration of Ab-01 activity. Since very small differences in the amount of RNA used in the amplification reaction can result in significant differences in C_T _values, the procedures for normalization of RNA amounts in starting reactions are described in detail here. The genes chosen as normalizer genes were *18S, GAPDH, GUSB, PGK*, and *ZNF592. (ZNF592 *was identified from a large GeneChip database as expressed at very consistent levels in human peripheral blood mononuclear cells, A. Hill and M. O'Toole, unpublished observations). The appropriateness of the 5 genes chosen as normalizers is demonstrated by the consistent expression among samples of each of the 5, Figure 1. RQ values were calculated using the delta delta C_T _method [12]. C_T _values of the endogenous controls were averaged for each sample. This average value was used to normalize RNA levels between samples. Expression levels of test genes were calculated as C_T _of gene - C_T _of the average of endogenous controls for that sample (delta C_T_). Gene expression values were calculated as: delta C_T _of gene minus delta C_T _of calibrator (delta delta C_T_) Data were then expressed as RQ (fold change over calibrator, 2E-delta delta C_T _or 2^-ΔΔC^_T_).

### Cynomolgus monkey PD biomarker assay development animals and sample collection

Adult cynomolgus monkeys (*Macaca fascicularis*; Charles River BRF, Inc, Houston, TX) weighing 6 to 9 kg were singly or pair housed and cared for according to the American Association for Accreditation of Laboratory Animal Care guidelines. The Wyeth Institutional Animal Care and Use Committee approved all aspects of this study. Under ketamine sedation (Ketaset, Fort Dodge Laboratories Inc., Fort Dodge, IA, 10 mg/kg IM), the femoral area was cleaned with povidone-iodine (Betadine; Purdue Frederick Co, Norwalk, CT) preparation solution followed by alcohol. Blood was drawn into Vacutainer CPT mononuclear cell preparation tubes (Catalogue #362761, BD, Franklin Lakes, NJ).

### *Ex vivo *treatment of monkey blood

rhIL-21 was added to aliquoted blood on the same day that the blood was drawn. When samples were treated with both antibody and rhIL21, the antibody was added and mixed thoroughly immediately prior to rhIL21 addition. Samples were then incubated at 37°C for 4 hours. Aliquots (0.5 mL) were removed and added to 2.0 mL microtubes (Axygen Scientific, #10011-744) containing 1.3 mLs of RNA*later*^® ^supplied with the Human RiboPure™-Blood Kit and mixed thoroughly by 5 complete inversions. Samples were stored at ambient temperature overnight and then frozen at -80°C pending RNA purification. This report and the report by Vugmeyster *et al. *[13] document the *ex vivo *response to rhIL21 stimulation in a combined total of 47 monkeys.

### Measurement of Ab-01 PD activity in monkeys dosed with Ab-01

Antibody was administered by means of bolus intravenous (i.v.) infusion via saphenous vein catheter (22G 1" Surflo, Terumo Co). Groups of animals were administered IgG_1_TM control antibody (n = 3), or Ab-01 (n = 3) at a dose of 10 mg/kg. Blood samples were drawn prior to antibody administration and 5 minutes post dosing.

### RNA isolation, description of custom TLDA and assay of RNA concentration for monkey studies

RNA isolation was performed as described above for the human blood assay. The pilot work for the assay was performed on the Human Immune TLDA (ABI), which contains assays measuring the levels of 96 different transcripts. Any assay that detected an IL21 response in human and/or monkey blood was selected for inclusion on a custom TLDA designed for the monkey studies. If the assay for the human gene was capable of measuring the monkey transcript, the human assay was retained on the custom TLDA for monkey studies. For genes that responded to IL21 in humans but were not detectable in the monkey using primers and probes designed for the human sequence, primers and probes designed to detect rhesus genes were used for the custom TLDA because the assay for cynomolgus monkeys were not, in general, available as predesigned Gene Expression Assays from ABI. All TaqMan assays included on the custom TLDA for monkey studies were among the "inventoried" assays available from ABI, and are described in Table 2. cDNA synthesis, preparation of samples for TLDA assay and measurements of RNA concentration were performed as described for the human assay.

**Table 2:** Assays used to measure monkey genes on custom TaqMan low density array for monkey studies

*Gene ID*	*Use*	*Species*	*Assay ID (ABI)*
*18S*	Manufacturing QC	Human	Hs99999901_s1
*CD19*	Effects of IL21/Ab-01	Human	Hs00174333_m1
*CSF1*	Effects of IL21/Ab-01	Human	Hs00174164_m1
*GUSb*	Normalizer	Human	Hs99999908_m1
*GZMB*	Effects of IL21/Ab-01	Rhesus	Rh02621701_m
*ICOS*	Effects of IL21/Ab-01	Rhesus	Rh02621771_m1
*IFN*_*γ*_	Effects of IL21/Ab-01	Rhesus	Rh02621721_m1
*IL10*	Effects of IL21/Ab-01	Human	Hs00174086_m1
*IL12B*	Effects of IL21/Ab-01	Human	Hs00233688_m1
*IL21R*	Effects of IL21/Ab-01	Human	Hs00174086_m
*IL2RA*	Effects of IL21/Ab-01	Human	Hs00166229_m1
*IL6*	Effects of IL21/Ab-01	Rhesus	Rh02621719_u1
*IL7*	Effects of IL21/Ab-01	Human	Hs00174202_m1
*IL8*	Effects of IL21/Ab-01	Human	Hs00174103_m1
*PGK1*	Normalizer	Human	Hs99999906_m1
*PRF1*	Effects of IL21/Ab-01	Human	Hs00169473_m1
*STAT3*	Effects of IL21/Ab-01	Human	Hs00234174_m1
*TBX2*	Effects of IL21/Ab-01	Human	Hs00203436_m1
*TNF*	Effects of IL21/Ab-01	Human	Hs00174128_m1
*ZNF592*	Normalizer	Human	Hs00206029_m1

In addition to the assays listed, assays for *CCL19, CSF2, IL-17*, and *REN *were run but found to be unreliable in these cynomolgus monkey samples and are not shown.

## Results

### Time course and dose response of *ex vivo *response of human whole blood to rhIL21

**Figure 2:**
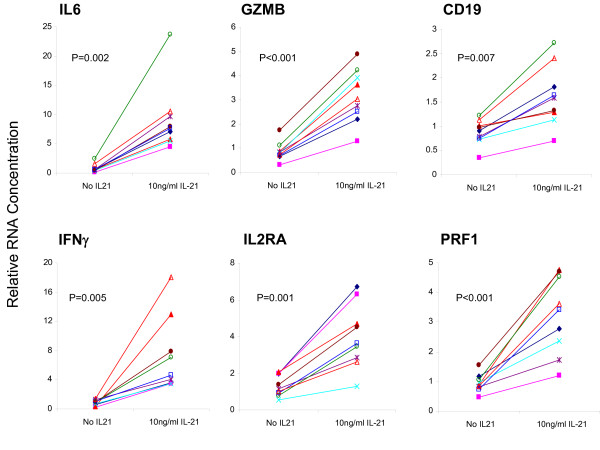
**Whole human blood response to *ex vivo *stimulation with 10 ng/mL rhIL21 for 2 hours**. Data shown are for the 6 genes (of 19 tested) with the most consistent response among the 9 individual donors, and are identified as 6 preferred biomarkers of rhIL21 activity in whole human blood. These 6 genes were also identified as the best indicators of IL21 response in a series of pilot studies conducted with blood from a different group of donors (data not shown).

**Figure 3:**
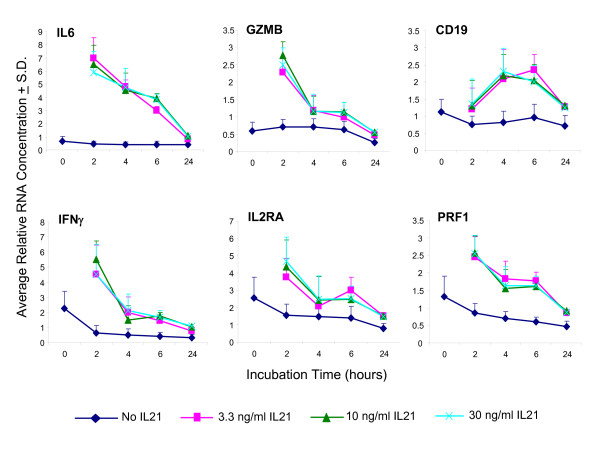
**Determination of optimal time point for rhIL21-response for the 6 genes identified as preferred biomarkers**. This time point experiment was done using 5 of the donors shown in Figure 2. Earlier pilot studies had suggested that stimulation for 30 minutes was significantly sub-optimal. There was no significant difference in response between all doses tested (30, 10, and 3 ng/mL). The lowest dose of rhIL21 that elicits a response has therefore not been determined.

Whole blood samples from 5 healthy human donors were incubated in the presence of 3.3, 10 or 30 ng/mL of rhIL21 for 2, 4, 6 or 24 hours. Consistent with prior pilot exploratory studies performed on 10 human blood donors, the most significant and robust rhIL21-dependent signals were obtained for six genes: *IL6, IFN*_*γ*_, *IL2RA, GZMB, PRF1, CD19 *(Figure 2). These 6 genes were therefore chosen as biomarkers of IL21 activity in whole blood. The optimal signal for all but *CD19 *was obtained at 2 hours (Figure 3). There was no difference in the response obtained at 3.3, 10 or 30 ng/mL rhIL21. Based on the results obtained with these 5 donors, the assay conditions chosen for subsequent experiments on *ex vivo *whole blood response to rhIL21 were 2 hour stimulation with 10 ng/mL of rhIL21.

### Titration of Ab-01 inhibition of *ex vivo *response to rhIL21

**Figure 4:**
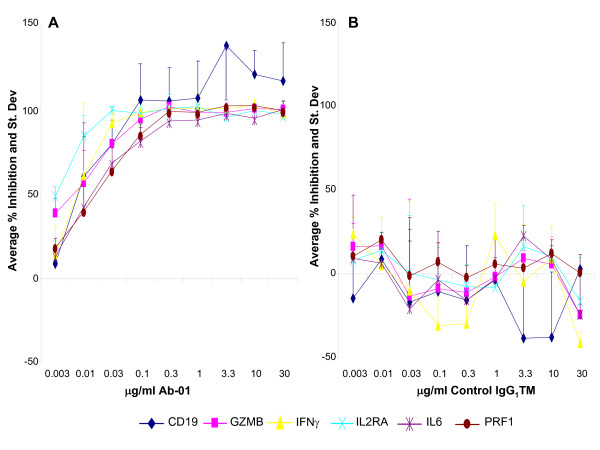
**Average percent inhibition of the expression level of 6 IL21-responsive genes**. Percent inhibition values were calculated based on RQ (relative quantification) values of untreated control and rhIL21-treated samples for each of the 4 donors, and subsequently the mean and standard deviation were determined for each gene shown. A: Percent inhibition in presence of Ab-01. B: Percent inhibition in presence of control IgG_1_TM. Data for the 0.1 μg/mL , 0.3 μg/mL and 1 μg/mL concentrations were generated using 4 donors. Data for the higher and the lower concentrations were generated using 2 donors.

**Figure 5:**
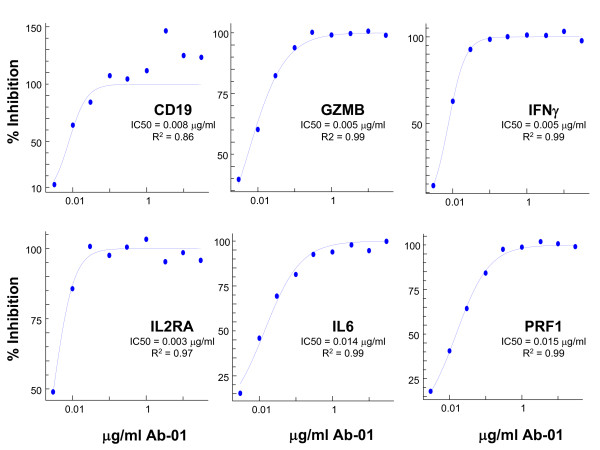
**Inhibition by Ab-01 at the indicated concentration is shown for 6 IL21-responsive genes**. IC50 values of inhibition curves shown in Figure 4A were calculated using curve fit (XLfit) program for each of the referred biomarker genes. Values for the 0.1 μg/mL, 0.3 μg/mL and 1 μg/mL concentrations were generated using 4 donors. Data for the higher and the lower concentrations were generated using 2 donors each.

Samples from 4 individual healthy human donors were pre-incubated for 2 hours at the indicated concentration of Ab-01 or the control IgG_1_TM prior to addition of 10 ng/mL rhIL21 and 2 hr incubation, and the effect on the 6 biomarkers was then assessed (Figures 4 and 5). For the first 2 donors tested, even the lowest concentration of Ab-01 (0.1 μg/mL, 0.66 nM) resulted in complete inhibition of the rhIL21 response, therefore the two subsequent donors were tested at increasing concentrations of Ab-01 starting at 0.003 μg/mL. Ab-01 inhibited the response of all 6 genes in all 4 donors. IC50 values ranged between 0.003 and 0.015 μg/mL Ab-01(Figure 5). Control IgG_1_TM had no significant effect on rhIL21 response (Figure4B).

### Ab-01 blocks signal transduction through cynomolgus rhIL21R

In order to determine if cynomolgus monkey was a suitable choice for safety studies, we tested whether the activity of rhIL21 on monkey cells was blocked by Ab-01. We therefore first examined the activation effects of *ex vivo *rhIL21 stimulation on cynomolgus blood cells, and observed very similar results to those observed with human blood. Results for 5 genes most significantly increased in monkey blood stimulated *ex vivo *with rhIL21 are shown in Table 3. The most robust (largest magnitude change and most consistent change) rhIL21-mediated change in cynomolgus monkeys was observed for *IL2RA*. All animals tested (n = 48) for effects of rhIL21 on *IL2RA *expression levels gave a response of >1.5 fold change [13], and therefore this gene was selected as the biomarker for subsequent monkey studies. To determine if Ab-01 had the desired blocking activity of rhIL21/IL21R dependent activation of cynomolgus blood cells, its ability to block rhIL21-dependent *IL2RA *activation was tested. The *IL2RA *response was effectively blocked by Ab-01 treatment (Table 4). There was no significant difference between the response to rhIL21 in the presence and absence of control IgG_1_TM, while the response in the presence of Ab-01 was blocked (P < 0.001). These results show that signal induction through the interaction of cynomolgus IL21R with rhIL21 is inhibited by Ab-01, an antibody to **human **IL21R. Therefore Ab-01 has the intended activity in cynomolgus monkeys.

**Table 3:** rhIL21-responsive genes in whole cynomolgus monkey blood

Gene	Average Fold Change (with IL21)	SD	P-value paired t-test
*IL2RA*	4.1	1.76	< 0.0001

*PRF1*	1.8	0.78	<0.0001

*IL21R*	2.3	0.63	<0.0001

*GZMB*	1.6	0.39	<0.0001

*IL6*	2.7	1.72	0.0007

rhIL21-mediated average fold increase in RNA expression in blood of 18 individual cynomolgus monkeys stimulated *in vitro *for 4 hours. Control (no rhIL21) RNA expression values were normalized to 1, and p values were calculated using log_2 _of the fold change paired with the log2 of 1 (0).

**Table 4:** Inhibition by Ab-01 of *ex vivo *rhIL21-dependent *IL2RA *expression

Animal	rHuIL21 alone	rHuIL21 + Control IgG_1_TM	rHuIL21 + Ab-01
1	4.7	3.5	1.2

2	3.8	3.8	0.9

3	4.1	5.1	1.4

4	2.8	2.8	0.9

5	3.3	3.2	1.3

6	2.8	2.9	1

7	3.6	3.7	1.1

8	7.2	8.8	1.1

Each value represents the fold change over the no-treatment group in expression levels of *IL2RA *in monkey blood samples following 4 hour *ex vivo *treatment as indicated.

### rhIL21-mediated activation is blocked in blood from monkeys dosed with Ab-01

The utility of the *ex vivo *rhIL21 stimulation assay as a read-out of Ab-01 PD activity in treated individuals was tested by comparing the rhIL21 response in 3 monkeys before and 5 minutes following administration of a 10 mg/kg i.v. dose of Ab-01. The control group consisted of 3 monkeys dosed with 10 mg/kg i.v. dose of IgG_1_TM. Consistent with the results from the many other monkeys tested, prior to dosing with Ab-01 an increase in *IL2RA *expression was observed in the blood of all 6 monkeys when stimulated *ex vivo *with rhIL21 (Table 5). No *IL2RA *response was observed in blood drawn 5 minutes following i.v. administration of Ab-01 (Table 5). Dosing with control IgG_1_TM did not affect the subsequent *ex vivo *response to rhIL21. Additional blood samples were tested at later time points following the single dose of Ab-01. Results showed that as the circulating levels of Ab-01 fell over time, the *ex vivo *rhIL21-mediated response was restored [13]. These results, together with the pre-dose and 5 minute post-dose data in Table 5 establish that all three monkeys dosed with Ab-01 were responsive to rhIL21 before dosing, did not respond when Ab-01 was present in the blood, and returned to responsiveness when Ab-01 was cleared from circulation.

**Table 5:** rhIL21 induced *IL2RA *expression in whole blood from cynomolgus monkeys dosed with Ab-01

Animal	Treatment Group	Time point	*Ex vivoIL2RA *Fold Change Response to IL21 Stimulation	C_peak_
1	Ab-01	pre-dose	2.8	not applicable

1	Ab-01	5 minute post dose	0.8	200

2	Ab-01	pre-dose	4.8	not applicable

2	Ab-01	5 minute post dose	0.8	139

3	Ab-01	pre-dose	4.2	not applicable

3	Ab-01	5 minute post dose	0.9	153

4	Control IgG_1_TM	pre-dose	4.2	not applicable

4	Control IgG_1_TM	5 minute post dose	4.3	not applicable

5	Control IgG_1_TM	pre-dose	2.7	not applicable

5	Control IgG_1_TM	5 minute post dose	4.4	not applicable

6	Control IgG_1_TM	pre-dose	9.1	not applicable

6	Control IgG_1_TM	5 minute post dose	7.7	not applicable

Relative RNA concentration of *IL2RA *induced by *ex vivo *addition of rhIL-21 to whole blood obtained from cynomolgus monkeys before and after treatment with either Ab-01 or control IgG_1_TM is shown. Pre-dose whole blood sample was taken on day -13 for animal 1, and on day 0 (the day of dosing) for all others. All monkeys were dosed with 10 mg/kg via intravenous (IV) route. The *ex vivo *response to IL21 was completely blocked in monkeys treated with Ab-01 and was not blocked in control IgG_1_TM treated monkeys

## Discussion

We have developed a human blood biomarker assay that detects the blocking activity of Ab-01, an antibody to IL21R. In parallel we have developed an adaptation of this assay and used it to demonstrate that the IL21-dependent response in cynomolgus monkey blood is blocked both by *ex vivo *addition of Ab-01 to blood, and by i.v. administration prior to blood collection. These results support the use of cynomolgus monkeys for safety studies by establishing that Ab-01 hits its target *in vivo *and has the desired biological activity in the species. We have shown that this assay system is well suited to examining the relationship between pharmacokinetics and pharmacodynamics (PK/PD), the intended clinical use of the assay system [13]. A third critical contribution, demonstrating lack of Ab-01 agonistic activity even under circumstances designed to force an agonistic signal is described in Guo et al [14]. The data reported here and in these related reports on Ab-01 are unified by their focus on addressing central challenges of translational medicine - dose selection, elucidation of PK/PD relationships, choice of safety study species, and mitigation of risk of immunotoxicity.

The assay reported here relies on an *ex vivo *stimulation procedure. It is difficult to develop a robust assay for determining Ab-01 PD activity by relying upon the endogenous levels of IL21 activity on biomarker gene expression in samples upon collection, especially in healthy donors. It is possible that such a strategy could correlate PK parameters and biomarker movement during the treatment period, but it would not show direct linkage between the movement of the biomarker and the engagement of the target. Following the biomarker assay development strategy previously employed by others, we have developed a whole blood *ex vivo *stimulation assay. Our biomarker discovery and assay development strategies have, from the start, proceeded with the realities of the clinical setting in mind. The volume of blood required is less than 5 mL, well below the limits of a routine blood draw. The live blood sample is subjected to minimal manipulation, consisting of separation into two aliquots, addition of rhIL21 to one of the aliquots, and 2 hour incubation at 37°C with rotation. RNA preserving solution is then added, and the sample frozen. All subsequent procedures can be carried out on batched samples in a validated lab. The assay is sensitive and robust, and has shown consistent performance among healthy human and primate subjects. We have shown that the assay detects Ab-01 in the 100 pM range in humans, well within the range for potential clinical utility.

We believe that the RNA assay system reported here has significant advantages over a protein secretion assay. First, the read-out of RNA is a more proximal event than protein secretion, and we have found that, in this assay system at least, the RNA signal is more easily and reliably detected than the protein signal [14]. Detection of protein secretion required a much longer incubation period, necessitating a shift from an assay with whole blood to an assay requiring purification and culturing of peripheral blood mononuclear cells. Such sample manipulation steps compromise the ease of adaptation for clinical use and introduce additional sources of variability. Secondly, measurements made on well purified RNA are not subject to the factors (such as for example, specific binding factors, charged proteins, or rheumatoid factor), which can confound ELISA assays performed in human serum or plasma [15]. We have also found that the cost of RNA assay systems compares favorably with that of ELISA systems, especially with considerations of standardization methodology.

Concordance between humans and cynomolgus monkeys with respect to genes activated was observed for *IL2RA, GZMB, IL6 *and *PRF1. IFN*_*γ *_also increases dramatically in both species, but detection of this increase in cynomolgus monkeys required a protocol modification (purification of PBMCs to increase *IFN*_*γ *_RNA yield) that was not performed for the 18-monkey cohort shown in Table 3. While *CD19 *is among the genes listed as most significantly increased by rhIL21 in humans, its absence from the list of genes in cynomolgus monkeys does not reflect a difference between the species, but rather the unavailability of a reliable TaqMan assay for *CD19 *in cynomolgus monkeys. A significant rhIL21-mediated *IL21R *elevation was observed in both humans and monkeys. The presence of *IL21R *on the top gene list for monkeys and not for humans reflects a higher relative ranking in monkeys due to the lack of *IFN*_*γ *_and *CD19 *data in cynomolgus monkeys (for the reason detailed above). We conclude that the whole blood rhIL21 responses of human and monkey are remarkably similar, and our work does not identify differences between them.

The goal of this work was to identify markers to be used to assess the PD activity of Ab-01 in treated subjects. For this reason, the most reliable read-outs detectable in whole blood were selected as the most useful from a clinical operations perspective. IL21 exerts many different effects on a wide variety of lymphoid and non-lymphoid cells including fibroblasts and intestinal epithelial cells [16-18]. The activities of IL21 have been shown to be highly dependent on the lineage of the target cell, the activation state of the target cell and the presence of other co-stimulators and immune modulators [19,20]. Previous studies have shown that IL21 activates both IFN_γ _and *IL2RA *in purified NK and purified T cells [21]. *CD19 *and *IL21R *were also among the genes that changed significantly upon *ex vivo *exposure of whole blood to rhIL21, and IL21 has been shown to up regulate both *CD19 *and *IL21R *expression in activatec B cells [22]. Therefore the markers identified in this study include genes with previously demonstrated links to activation of the IL21 pathway in a variety of cell types. In light of the well established complexities of IL21 biological activity, it is fortuitous from a clinical development perspective that whole blood provides a useful analytical sample for detecting inhibition by a candidate therapeutic of signal transduction through IL21R.

## Conclusions

The PD biomarker assay described here has been developed to facilitate safe and efficient clinical testing by positioning a clinical team to make dose selection decisions based on reliable information on *in vivo *biological activity. The assay system monitors levels of activity in blood and will indicate when levels are too low to block activity and when levels are significantly higher than they need to be. The basic approach here is also applicable to other therapeutic candidates, especially in indications related to inflammation. The demonstration of the desired biological activity in the safety study species is also a significant contribution in preparing for transition to the clinic.

## Competing interests

All authors were employees of Pfizer (formerly Wyeth) at the time this work was performed.

## Authors' contributions

MA performed all the studies on human samples reported here, analyzed the data and co-wrote the manuscript. SJ and AAW performed all the pilot studies in humans and monkeys that identified the candidate biomarkers, and they participated in the data analyses. AAH developed the customized Spotfire tool used for data analyses and reviewed statistical analyses. YG participated in the assessment and selection of TaqMan PCR assays for studies in monkeys and participated in the biomarker discovery (pilot) phase of the project. AGB performed all in vivo procedures on monkeys and contributed to the writing of the manuscript. MFS was responsible for interface with the clinical team and for securing and scheduling the resources that will be required upon hand-off for assay validation. SJ, AW, SWA and KA performed the *in vitro *assays on monkeys treated with Ab-01 and control Ig and analyzed the data, and KA and SA performed the experiments on monkeys dosed in vivo with Ab-01 or IgG_1_TM. ERL performed data analysis and review and participated in the drafting of the manuscript. DY co-led the Ab-01 team and was responsible for studies related to the characterization of the biological properties of the antibody. LB co-led the Ab-01 team and was responsible for lead antibody selection and development. MO devised the PD assay strategy, directed the biomarker discovery process, supervised assay development and co-wrote the manuscript. All authors have read and approved the final manuscript.

## Supplementary Material

Additional file 1 **Description of rhIL21**. Sequence and information on preparation and activity of the rhIL21 protein preparation used in these studies is shown. Click here for file
