# Supplementary material for: Development and application of a biomarker assay for determining the pharmacodynamic activity of an antagonist candidate biotherapeutic antibody to IL21R in whole blood
Source: J Transl Med. 2010 May 28;8:51. doi: 10.1186/1479-5876-8-51 (PMC2892437; doi:10.1186/1479-5876-8-51)
Supplement: Additional file 1 — Description of rhIL21. Sequence and information on preparation and activity of the rhIL21 protein preparation used in these studies is shown. [file 1479-5876-8-51-S1.DOC]

### Additional file 1 – Description of rhIL21

**Recombinant human IL21 cytokine, n-terminal His with enterokinase site**

HHHHHH**GSGDYKDDDDKGS**GQDRHMIRMQLIDIVDQLKNYVNDLVPEFLPAPEDVETNCEWSAFSCFQKAQLKSANTGNN

ERIINVSIKKLKRKPPSTNAGRRQKHRLTCPSCDSYEKKPPKEFLERFKSLLQKMIHQHLSSRTHGSEDS

This protein has been tested in IL21-dependant engineered and primary cells. In cell based assay it was found to be active in stimulation of cell growth and activity was specifically neutralized by the receptor extra-cellular domain, IL21R-Fc, or anti-IL21R antibodies. Activity has been confirmed *in vivo* in the air pouch model, shown to trigger white blood cell infiltrates, and activity neutralized by IL21R-Fc.
